# Mental Maladies—A Treatise on Insanity

**Published:** 1845-08

**Authors:** 


### BIBLIOGRAPHICAL NOTICES.

*Mental Maladies. A Treatise on Insanity.* By E. ESQUIROL, Physician-in-Chief of the Maison Royale des Aliénés de Charenton, &c. Translated from the French, with additions, by E. K. Hunt, M. D. Philadelphia: Lea & Blanchard, 1845. pp. 496. (From the publishers.)

The author's name is sufficient, alone, to recommend this work to the profession, and it is scarcely necessary to do more therefore, than simply announce an American edition of it. We may add that the style is lively and pleasing in the extreme, the descriptions vivid and faithful, the views for the most part just and broad, and it is pervaded at once by a spirit of philanthropy and love of the subject treated, which renders it a most interesting book even to the general reader. The Journal of Insanity, a competent authority, pronounces this the best of all the works that have appeared upon these diseases.

The principle of classification adopted is that of the ancients, dividing all mental diseases into five classes, viz: 1. Lypemania (Melancholy); 2. Monomania; 3. Mania; 4. Dementia; 5. Idiocy. It is known that other classifications have lately been recommended, particularly that which is based upon the different classes of faculties, as of perception, intellect, sentiment, &c., but this has not as yet been found so valuable as the former in its practical application to the treatment of the insane, whatever may be the merits of its principles considered abstractly.

It may be interesting to our readers to know what so eminent an observer as Esquirol thought of the pathology of Insanity, and of the functions of the different parts of the brain. In reference to the latter point the following quotation is offered:—"All the labor that has been expended on the anatomy of the brain, has produced no other result than a more exact description of this organ, and the despairing certainty of our being forever unable to assign to its parts, the uses from whence we may derive information, with reference to the exercise of the thinking faculty, whether in health or disease." It is only one who knows well, both the history of our knowledge of the brain, as well as the present state of it, who can appreciate the grounds on which the above opinion rests; which, however, we trust, is yet less encouraging than the facts would justify.

In regard to the former, the pathology of insanity, we offer the following more extended extract:

1. Vices of conformation in the cranium, are met with, only among imbeciles, idiots, and cretins.

2. Organic lesions of the encephalon and its envelopes, have been observed only among those whose insanity was complicated with paralysis, convulsions and epilepsy; or rather, these lesions appertain to the malady which has caused the death of the patients.

3. The sanguine or serous effusions; the injections, or infiltrations, which we meet with in the cranial cavity; the thickening of the meninges; their adhesions among themselves, with the cranium and the gray substance; the partial or general softening of the brain; the density of this organ; the fibrous, knotty, and cancerous tumors, observed within the cranium; all these alterations indicate either the causes or effects of insanity; or rather the effects of a complication to which the patients have yielded.

4. The alterations within the thorax, abdomen and pelvic cavity, are evidently independent of insanity. These alterations may, nevertheless, indicate the source of mental alienation, by showing the organ primitively affected, which has reacted upon the brain.

5. All the organic lesions observed among the insane, are found to exist among those, who have never suffered from chronic delirium.

6. Many post-mortem examinations of the insane, have revealed no alteration, although the insanity may have persisted for a great number of years.

7. Pathological anatomy, shows us every part of the encephalon, altered, in a state of suppuration, and destroyed, without chronic lesion of the understanding.

8. From the above data, we may conclude, that there are cases of insanity, whose immediate cause escapes our means of investigation; that insanity depends upon an unknown modification of the brain; that it has not always its point of departure in the brain, but rather in the foci of sensibility, situated in different regions of the body; as disorders of the circulation do not always depend upon lesions of the heart, but upon those of some other portion of the vascular system.

The plan of treatment recommended is "isolation," or the removal of the patient from all ordinary scenes and associations, together with such medical treatment as each case may require. If we duly consider the influence of surrounding circumstances upon the mind, that they mould the character, and in certain cases, as in solitary confinement, efface the intellectual and moral faculties, we shall be prepared to understand the effect with which they are employed in the treatment of these diseases. While recoveries are comparatively rare among those treated at their own houses, or in private houses, one-half of those sent to well regulated retreats, are cured. We will add, in conclusion, that a portion, only, of the work of Esquirol has been translated by Dr. Hunt. All that part which relates to the statistics and hygiene of establishments for the insane, and the medico-legal relations of the subject, has been omitted. D. B.

